# Self-renewal and phenotypic conversion are the main physiological responses of macrophages to the endogenous estrogen surge

**DOI:** 10.1038/srep44270

**Published:** 2017-03-20

**Authors:** Giovanna Pepe, Daniele Braga, Tiziana A. Renzi, Alessandro Villa, Chiara Bolego, Francesca D’Avila, Cristina Barlassina, Adriana Maggi, Massimo Locati, Elisabetta Vegeto

**Affiliations:** 1 Center of Excellence on Neurodegenerative Diseases and Department of Pharmacological and Biomolecular Sciences, University of Milan, Italy; 2 Department of Health Sciences, University of Milan, Italy; 3 Fondazione Filarete, Milan, Italy; 4 Department of Medical Biotechnologies and Translational Medicine, University of Milan, Italy; 5 Humanitas Clinical and Research Center, Rozzano, Italy; 6 Department of Pharmaceutical Sciences, University of Padua, Italy

## Abstract

Beyond the physiology of reproduction, estrogen controls the homeostasis of several tissues. Although macrophages play a key role in tissue remodeling, the interplay with estrogen is still ill defined. Using a transcriptomic approach we first obtained a comprehensive list of genes that are differentially expressed in peritoneal macrophages in response to physiological levels of 17β-estradiol (E_2_) injected in intact female mice. Our data also showed the dynamic nature of the macrophage response to E_2_ and pointed to specific biological programs induced by the hormone, with cell proliferation, immune response and wound healing being the most prominent functional categories. Indeed, the exogenous administration of E_2_ and, more importantly, the endogenous hormonal surge proved to support macrophage proliferation *in vivo*, as shown by cell cycle gene expression, BrdU incorporation and cell number. Furthermore, E_2_ promoted an anti-inflammatory and pro-resolving macrophage phenotype, which converged on the induction of genes related to macrophage alternative activation and on IL-10 expression *in vivo*. Hormone action was maintained in an experimental model of peritoneal inflammation based on zymosan injection. These findings highlight a direct effect of estrogen on macrophage expansion and phenotypic adaptation in homeostatic conditions and suggest a role for this interplay in inflammatory pathologies.

Macrophages are key cells in inflammation and immunity and can adopt different activation states depending on the activating signal and context. Schematically, inflammatory macrophages adopt a classical (M1) activation phenotype, which is mimicked *in vitro* by exposure to interferon-γ and toll-like receptor ligands, characterized by the synthesis and/or secretion of cytotoxic and chemotactic mediators that promote inflammation and the killing of pathogens and tumors. Immunoregulatory signals produced by surrounding cells, including interleukin-4 (IL-4) and IL-13, or by activated macrophages, namely IL-10 and TGFβ, stimulate alternative programs of phenotypic activation which promote resolution of inflammation, tissue repair, and homeostasis restoration, frequently alluded to as M2. M1 and M2 activation states are two extremes of a range of intermediate phenotypes that macrophages can acquire through their unique ability to integrate a vast array of signals under the constraint of environmental factors. The macrophage phenotype is also influenced by systemic endogenous molecules that tune their ability to respond to danger signals, as exemplified by glucocorticoid hormones^1^. Thus, different activation profiles provide reactive macrophages with immune and metabolic properties that address them towards specialized tasks sustained by distinct gene expression programs^2^.

Estrogen hormones are lipophilic molecules with pleiotropic functions including a well-documented influence on the inflammatory process, as revealed by the variation of chronic inflammatory diseases occurrence and response to infections in relation with the menstrual cycle, pregnancy and menopause^3^,^4^. Estrogen effects on immune activity are also revealed by the higher rate of autoimmune diseases in women when compared to men, and by the strict dependence of symptomatology depending on women’s estrogen levels^5^. Experimental models have shown that the development of innate immune cells and the immune responses to danger signals are highly influenced by estrogen, converging on an anti-inflammatory effect of this hormone in dose and context-dependent manners^4^,^6^. Accordingly, animal models of inflammation are highly responsive to estrogen insufficiency and replacement.

Estrogen action is mediated by its interaction with estrogen receptors (ERs), ERα and ERβ, which operates as hormone-regulated transcription factors as well as through non-genomic effects, such as calcium influx or lipid kinase activation, also mediated by a more recently described G protein-coupled estrogen receptor, GPR30. ERs and GPR30 were shown to be expressed by human and mouse macrophages and to influence inflammation, with ERα being one key mediator of the estrogen anti-inflammatory activity in these cells^6^,^7^,^8^,^9^,^10^. While the impact of estrogen and macrophage physiology is gaining intriguing insights for both endocrine and immune implications and for human pathologies^11^,^12^, our knowledge of the transcriptional response of macrophages to this hormone is limited to the effect of the hormone on specific molecular targets, such as VEGFα or inflammatory cytokines, in cells exposed to specific immunologic ligands^13^,^14^,^15^. The aim of this study was to provide a comprehensive description of the transcriptional response of macrophages to this hormone evaluating the genomic and functional response of macrophages exposed *in vivo* to physiological changes in estrogen levels.

## Results

### Gene expression profiling of peritoneal macrophages in response to estrogen *in vivo*

**Figure 1:**
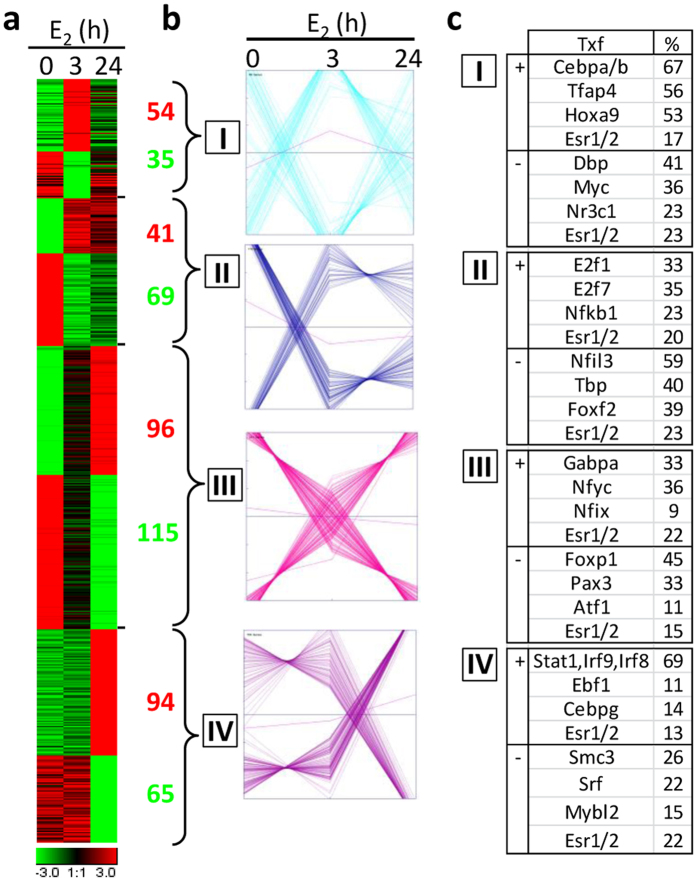
The transcriptome analysis of peritoneal macrophages in response to estrogen. Non-elicited peritoneal macrophages obtained from metaestrous (ME) female mice were analyzed by RNA sequencing after the *in vivo* treatment with vehicle (time 0, n = 3) or E_2_ administration for 3 (n = 3) or 24 h (n = 3). (**a**) The temporal expression profile of DEGs is represented as heat map (red, high relative expression; black, mean expression; green, low relative expression). (**b**) By k-means clustering calculations, DEGs were grouped in 4 clusters according to the absolute variation in their expression profile. The number of up (red) and down (green)-regulated genes in each cluster is shown. Gene expression levels are shown in relation to the mean expression value of the DEGs in each cluster (magenta line). (**c**) Transcription factors (Txf) that bind putative binding sites in up (+) or down (−)-regulated genes are listed for each cluster; the column on the right shows the number of genes with Txf binding sites in their promoters reported as the percentage of the total number of up (+) or down (−)-regulated genes in each cluster. Esr1/2: binding sites for estrogen receptors^16^.

ERs are ligand-dependent transcription factors that induce genomic responses by the direct interaction with target gene promoters as well as through indirect secondary mechanisms. Since peritoneal macrophages were shown to express ERs^13^,^15^, we envisioned to analyze the transcriptomic response to estrogen of peritoneal macrophages. Female mice were selected in the metaestrous (ME) phase of the estrous cycle, bearing low levels of circulating estrogens, and either left untreated or injected with a physiological dose of E_2_. This approach provides an experimental asset to more faithfully mimic the mechanisms induced by the physiological estrogen surge in intact animals. After 3 or 24 h of hormone injection, peritoneal macrophages were purified by immunosorting, RNA extracted and analyzed by RNA sequencing. Our data show that short and long term hormonal treatments significantly affected expression levels of 569 transcripts in total. When data were analyzed grouping up- or down-regulated differentially expressed genes (DEGs) according to onset and duration of hormone action considered as net effect, four gene clusters could be identified: cluster I) collects DEGs related to early and transient effects (only regulated at 3 h); cluster II) gathers DEGs supporting persistent responses (similarly modulated by 3 and 24 h treatments); cluster III) includes early and progressively responsive genes (regulated at 3 h and furthermore at 24 h treatment); cluster IV) contains late responsive genes (only influenced after 24 h treatment) (Fig. 1a). Analysis of clusters kinetics showed that a significant fraction of DEGs (35%; clusters I + II) are already modulated 3 h after E_2_ administration thus behaving as early responsive genes. Only a minor fraction of them returned to control levels after 24 h treatment (16%; cluster I), while the majority of all DEGs are modulated at this late time point (84%; clusters II + III + IV). Progressively responsive genes represent the highest number of regulated genes (37%; Cluster III), while genes regulated only at late time point constitute a high proportion of genes (28%; cluster IV) (Fig. 1b). The list of differentially expressed genes (DEGs), ranked by their sensitivity to estrogen in comparative analyses, and its association with the respective cluster is shown in Supplementary Table 1.

Several transcription factors, including ERs, may participate in regulating DEGs expression. In order to gain insight into the mechanisms of hormone responsiveness, motif enrichment analysis was conducted to search for transcriptional regulatory elements in the promoters of macrophage DEGs. Each cluster included unique transcription factors that are involved either in the up- or down-regulation effects, with up-regulated genes in cluster I showing a high abundance of binding sites for C/EBP transcription factors, an enrichment for E2f binding sites in cluster II, and for Irf and Stat families of transcription factors in cluster IV (Fig. 1c). In parallel, specific transcriptional regulators are involved also in the down-regulation of DEGs in a cluster-specific manner. Analysis of ER binding sites^16^ shows cross-interference among different clusters, as this regulatory element is enriched with similar frequency of DEGs enhancers/promoters in all fours clusters (Fig. 1c and Supplementary Table 1). Thus, these analyses support the hypothesis that early and late effects induced by estrogen on macrophage transcriptome are mediated by direct ERE-mediated mechanisms as well as by the ordered engagement of distinct sets of transcription factors that allow the specific temporal profile of gene regulation observed in response to estrogen. Taken together, these data show that the *in vivo* administration of a physiological dose of E_2_ results in the regulation of expression of a distinct set of genes in macrophages, an effect which is evident shortly after hormone administration and progresses with time through a balanced combination of positive and negative distinct or overlapping pathways.

### Functional modifications associated with estrogen action in macrophages

**Figure 2:**
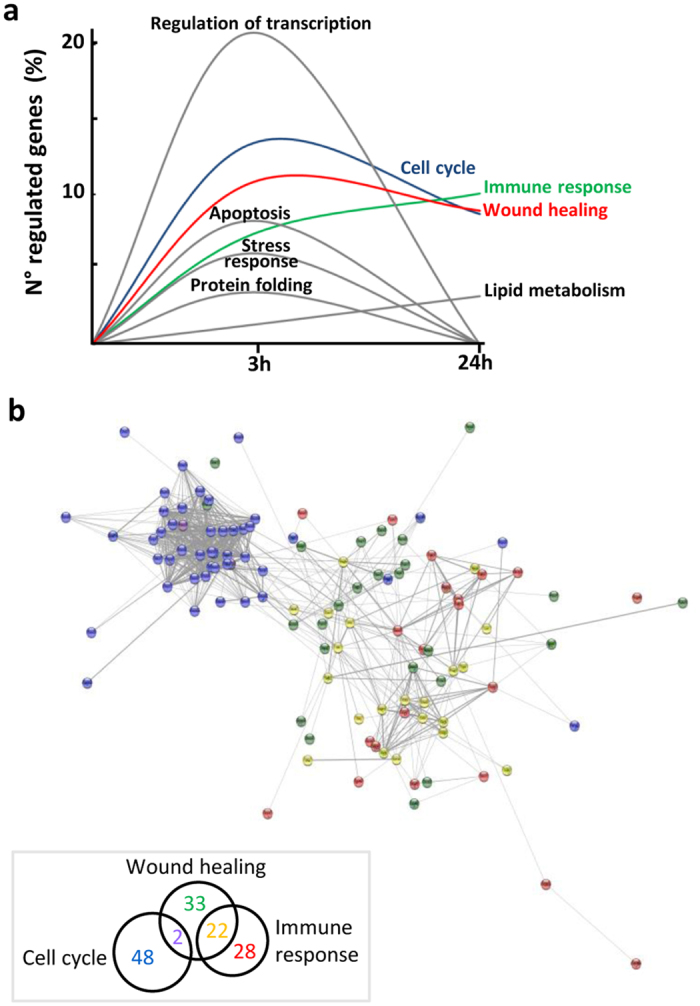
Biological pathways regulated by E_2_ in macrophages. (**a**) The DEGs list from RNA sequencing analysis was submitted to the DAVID database for functional annotation. GO ontologies significantly modulated following 3 or 24 h of estrogen treatment are represented, with the number of genes regulated in each ontology reported as % of all regulated genes at each treatment. *Immune response* includes Immune response, Chemotaxis and Endocytosis; *Wound healing* includes Response to wounding, Hemopoietic/lymphoid organ development and Blood vessel development; *Lipid metabolism* includes Positive regulation of lipid metabolic process, Cholesterol metabolic process and Lipid localization. Ontology gene lists are reported in Supplementary Table 2. (**b**) String diagram of the interactions among regulated genes. Genes of the Cell cycle (blue), Immune response (green) and Wound healing (red) are evidenced, with commonly regulated genes resulting in purple (Cell cycle + Immune response) and yellow (Immune response + Wound healing). The Venn diagram in the insert shows the numbers of unique or common regulated genes. (See Supplementary Figure 3 for a high resolution image of panel b).

In order to translate comprehensive genomic information into functional relevance, we performed functional annotation analyses using Gene Ontology term enrichment to search for significant terms related with biological programs. Enrichment of specific biologic categories (Supplementary Table 2) showed that, in analogy with gene expression, the time-course evaluation of estrogen action identifies biological programs that are either uniquely or commonly regulated as early or late responses to estrogen in macrophages (Fig. 2a). Genes showing early and transient response to estrogen resulted enriched in specific gene ontologies including transcription factors, apoptosis, stress response, and protein folding, while the ontology related with lipid metabolism was enriched as a late responsive pathway. However, three functional programs clearly emerged as persistent estrogen-responsive pathways in macrophages: these relate with cell cycle, immune response, and wound healing. As shown in Fig. 2b, a search for functional interactions using the String database resulted in a high number of connections among proteins encoded by genes involved in the *Cell cycle, Immune response* and *Wound healing* processes. The first category appears to be supported by the coordinated regulation of a core of highly interconnected genes, while the last two categories share a high number of proteins modulated by estrogen and are known to converge on the alternative polarization of macrophages (Fig. 2b). Interestingly, some of the transcription factors shown in Fig. 1c are known to be involved in cell cycle progression, including C/EBPs and E2fs or in the control of inflammation and macrophage polarization, including NF-kB, FOXP1 and Gabpa^17^, while some others were shown to regulate both processes, such as Myc^18^, further supporting the functional implications of estrogen action on macrophages and providing a first hint on the underlying molecular mechanisms. Altogether, these results show that estrogen induces in macrophages specific biological pathways related to distinct functional responses supported by an highly interconnected network of hormone-responsive genes.

### Estrogen modulates gene expression in isolated macrophages

**Figure 3:**
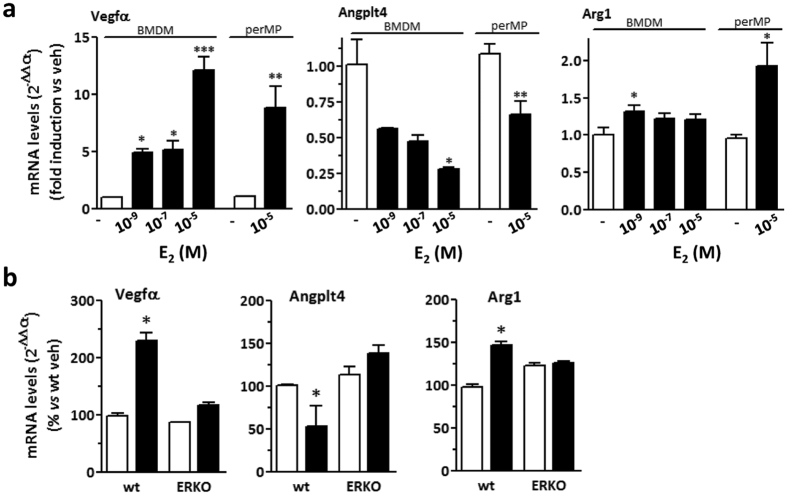
Regulation of target gene expression by E_2_ in isolated macrophages. Peritoneal macrophages (perMP) and BMDM were grown in culture and assayed for gene expression following E_2_ administration. (**a**) Macrophage cells were treated with vehicle (veh, open bars) or increasing concentrations of E_2_ (10^–9^, 10^−7^ and 10^−5^ M, filled bars) for 3 h. Real time PCR was used to analyze the mRNA levels coding for Vegfα, Angtpl4 and Arg1. (**b**) Comparison of estrogen action in BMDM cells from WT and ERα-KO (ERKO) mice. The mRNA levels coding for Vegfα, Angtpl4 and Arg1 were analyzed 24 h after the addition of vehicle (veh, open bars) or 10^−7^ M E_2_ (filled bars). Data sets for each gene were calculated using the 2^−ddCt^ method with respect to the mean value of the vehicle group. Bars represent mean values ± SEM (n = 4). *p < 0.05; **p < 0.01 versus veh.

Binding sites for ERs are present in the promoters of some of the DEGs in clusters I-IV. In order to ascertain that the transcriptional response of macrophages to estrogen was mediated by a direct effect of the hormone, macrophages were grown in culture and assayed for estrogen action on the expression of target genes, with or without EREs in their promoters. Peritoneal macrophages and BMDMs were cultured *ex vivo* and assayed for the expression of estrogen target genes in response to E_2_; since the peritoneal fluid in women may contain micromolar concentrations of ovarian hormones through exudation during ovarian follicle development and rupture, E_2_ concentrations higher than the nanomolar plasmatic range were also tested, although anatomical differences may lead to a reduction in estrogen concentrations in the female mouse peritoneum^19^,^20^. As shown in Fig. 3a, transcripts coding for Angtpl-4 and VEGFα, cluster I and II ERE-positive genes respectively, were modulated by increasing concentrations of estrogen in isolated macrophages. Interestingly, levels of transcript coding for Arg1, a cluster II gene with no ERE in its promoter/enhancer region, were also significantly increased by short-term estrogen treatment, though the overall effect of estrogen was significantly lower. The effect of hormone in isolated macrophages was similar to that observed *in vivo* with superimposable fold-inductions in the mRNAs analyzed (see Fig. 3 and Supplementary Table 1). To further validate the involvement of estrogen-specific signaling pathways, we evaluated hormone action on the genes reported above using macrophages from ERα-KO animals. Consistent with previous reports from this and other groups^6^,^8^,^21^,^22^ ablation of ERα gene resulted in loss of estrogen action in macrophages, as estrogen-induced changes in the mRNA levels coding for Vegfα, Angtpl4 and Arg1 observed in WT cells were not detected in ERα-KO macrophages (Fig. 3b). Altogether, these data suggest that estrogen acts directly on macrophages to induce a complex gene expression program mediated, at least in part, by the ERα receptor.

### Estrogen regulates the expression of genes associated to proliferation and macrophage polarization

**Figure 4:**
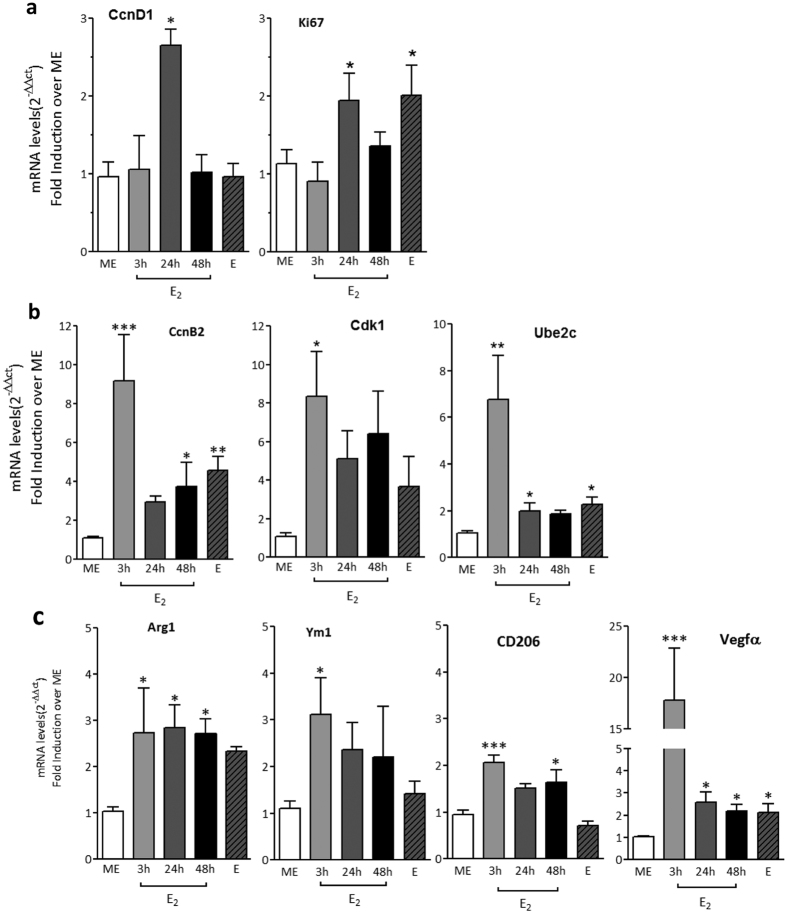
Validation of E_2_ target genes in peritoneal macrophages *in vivo*. Peritoneal macrophages were isolated from female mice at the metaestrous phase (ME) of the estrous cycle or treated with E_2_ for 3, 24 and 48 h. Females in the estrous phase (E) were also investigated. Real time PCR was performed to detect the mRNAs coding for genes related with cell cycle phases (**a**) G1/S (CcnD1, E2f1), (**b**) G2/M (CcnB2, Cdk1, Ube2c; Ki67) and (**c**) alternative polarization (Arg1, Ym1, CD206, Vegfα). Data sets for each gene were calculated using the 2^−ddCt^ method with respect to the mean value of the control group (ME). Bars represent mean values ± SEM (n = 5–10). *p < 0.05; **p < 0.01; ***p < 0.001 versus ME.

The identification of estrogen-responsive genes and the indication of specific biological pathways modified by estrogen suggest that macrophages may undergo functional changes in response to varying hormonal levels. Cell replication and polarization consistently emerged as hormone-responsive programs and were thus selected for deeper investigation. We thus set our next experiments to extend our observation by further modifying hormonal treatments and, more importantly, to evaluate the effects of changes in endogenous estrogen. As a first step, gene expression data obtained by RNA sequencing were validated using semi-quantitative analyses on peritoneal macrophages extracted from female mice at ME or following E_2_ treatments for 3, 24 and 48 h. In addition, a group of females in the estrous phase, which immediately follows the rapid physiological surge of estrogens, were also included in the analyses in order to evaluate macrophage gene expression in response to the endogenous increase in female hormones. Figure 4a and b show that mRNA levels of genes associated to actively replicating cells, such as Ki67 and Ube2c, and coding for proteins related with cell cycle phases, namely CcnD1 gene for the G1/S phase and CcnB2 and Cdk1 genes for the G2/M phase, were increased following the administration of exogenous estrogen. For all genes but Ki67 and CcnD1, the highest induction was reached after 3 h treatment followed by a substantial decrease at later time-points. Interestingly, the expression of some of these genes was also significantly increased in the estrous phase, showing that also the endogenous surge of female hormones is followed by an increased expression of cell cycle and replication genes in macrophages. Transcripts coding for proteins associated with macrophage polarization phenotypes, such as Arg1, Ym1, CD206 and VEGFα, were also upregulated by estrogen with the highest induction occurring shortly after hormone administration. Interestingly, for these genes the effect was still observed at extended time points (Fig. 4c). Arg1 and Vegfα mRNAs are also increased in macrophages from females at estrous, further demonstrating that the endogenous surge of female hormones is associated with an increased expression of genes that are related with the immune polarization of these cells. Taken together, these data demonstrate that genes coding for proteins involved in cell cycle progression and in M2 polarization are induced by an increase in either exogenous or endogenous estrogen levels.

### Increased estrogen levels promote macrophage proliferation *in vivo*

**Figure 5:**
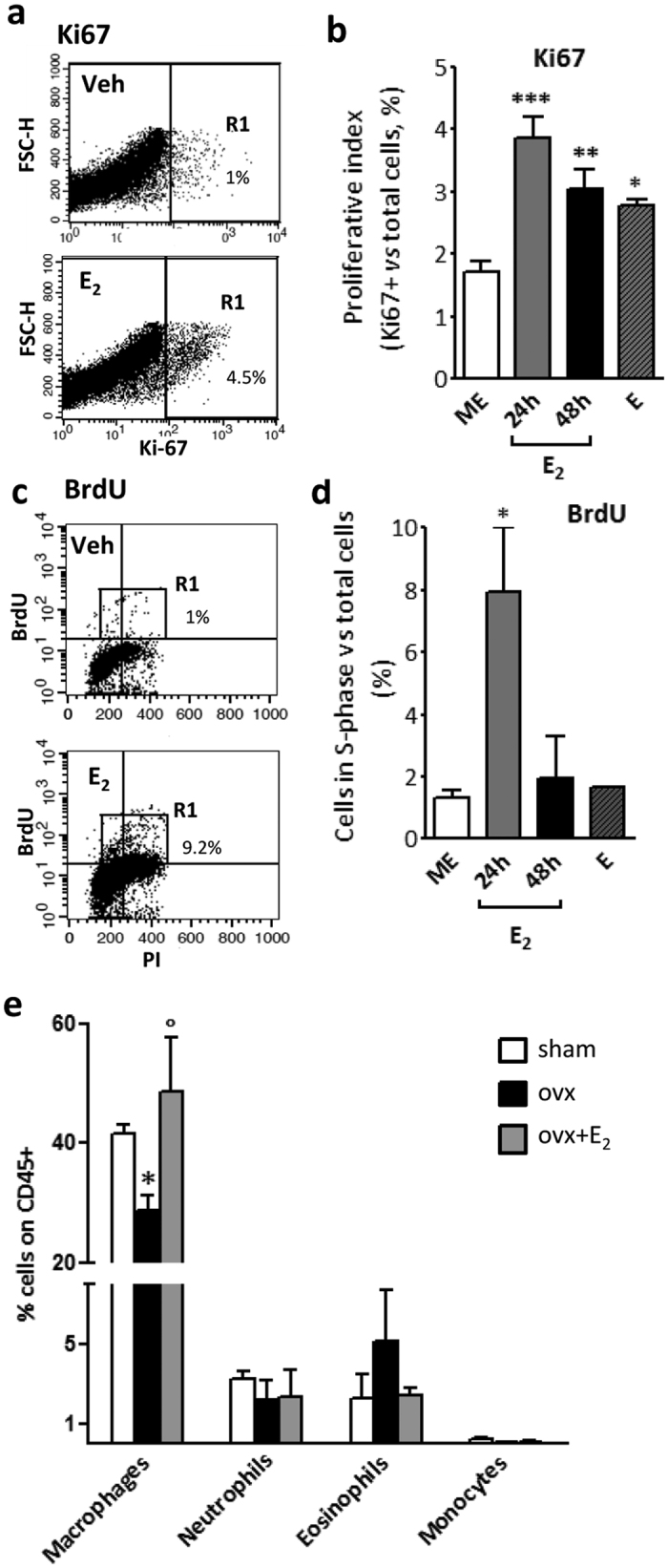
Estrogen induces macrophage proliferation. (**a**–**d**), Proliferation was analyzed by evaluating Ki67 expression and BrdU incorporation by peritoneal macrophages from female mice at ME or 24 and 48 h after E_2_ treatment. Females in the estrus phase (**e**) were also analyzed. Mice were injected i.p. with BrdU 2 h prior to analysis. Representative dot plots depicting gating schemes for Ki67 and BrdU analysis are shown in panels a and c, respectively. Doublets were removed based on FL2 scatter width (FL2-W)/FL2 scatter area (FL2-A). Bar charts show the percentage of Ki67 (**b**) and BrdU (**d**) positive macrophages. Data are representative of three independent experiments. Graphs represent the mean ± SEM of 4–9 mice per group. (**e**), Ovariectomized female mice were treated with vehicle (ovx, black bars) or E_2_ (grey bars) for 60 h. Sham-operated mice treated with vehicle were used as controls (open bars). The peritoneal lavage fluid was analyzed by flow cytometry to evaluate the percentage of macrophages and other immune cells, as indicated. Results are expressed as the mean ± SEM (n = 3). *p < 0.05 *versus* sham; °p < 0.05 *versus* ovx.

Peritoneal macrophages are maintained in steady state by a low rate self-renewal process^23^,^24^,^25^. As transcriptional profile data show that estrogen regulates a significant number of genes associated to proliferation while having a minor effect on inflammatory cytokines involved in leukocyte recruitment, we addressed the possibility that E_2_ acts as a proliferative signal for peritoneal macrophages. In agreement with its induction at the transcriptional level (Fig. 4a and b), a significant increase in the fraction of Ki67^+^ peritoneal macrophages was observed in animals treated for 24–48 h with E_2_ (Fig. 5a and b). Accordingly, females in the estrous phase have a higher number of Ki67^+^ macrophages cells in the peritoneum compared with ME females (Fig. 5b). Thus, these results suggest that an increase in circulating estrogen levels enhances the proliferation rate of peritoneal macrophages. To further extend this functional evidence, BrdU was administered *in vivo* 2 h before sacrifice and its incorporation into the DNA of peritoneal macrophages was assessed to quantify cells in the S-phase of the cell cycle. Figure 5c shows that a 24 h treatment with E_2_ significantly increased the percentage of cells that were duplicating their DNA. BrdU incorporation was not increased in the other experimental groups, suggesting that the effect on DNA duplication was restricted to a specific time window of E_2_ action encompassing the first 24 h after treatment without being detected in the estrous phase or at a later time point (48 h; Fig. 5d), when the increased number of cells progressing in the cell cycle (assessed by Ki67 expression in Fig. 5b) is probably too small to allow the detection of cells within the S-phase. Of note, the amplitude of estrogen action on BrdU incorporation is similar to that reported for IL-4, the best-characterized signal inducing local expansion of peritoneal macrophages in inflammatory conditions^26^.

As genomic data and functional evidence indicated that an increased number of macrophages in the S-phase of the cell cycle is present in the peritoneum of estrogen-treated female mice, we asked whether the number of peritoneal macrophages could be influenced by alterations in the endogenous levels of estrogen. Indeed, ovariectomized (ovx) female mice, in which production and biological effects of endogenous estrogen are ceased, showed a significant reduction in the number of peritoneal macrophages as compared to sham-operated animals, an effect that was completely reverted by a long-term exposure to exogenous E_2_ (Fig. 5e). Peritoneal macrophages are a heterogeneous population of cells, which includes large peritoneal macrophages (LPM), characterized by the expression of the transcription factor Gata6, and small peritoneal macrophages (SPM), a less abundant subset of macrophages that derive from circulating monocytes^23^,^24^,^25^. The abundance of LPS, SPM and other leukocyte populations, as well as the efficiency of cell recovery procedures were not altered by changes in estrogen levels (see Supplementary Fig. 2). Altogether, these data demonstrate that the endogenous or exogenous surge of estrogen enhances peritoneal macrophage proliferation. The proliferative signature of macrophages exposed to estrogen correlates with an increase in their number, in the absence of a significant increase in other leukocyte types.

### Estrogen promotes macrophage alternative polarization in an *in vivo* model of self-resolving inflammation.

**Figure 6:**
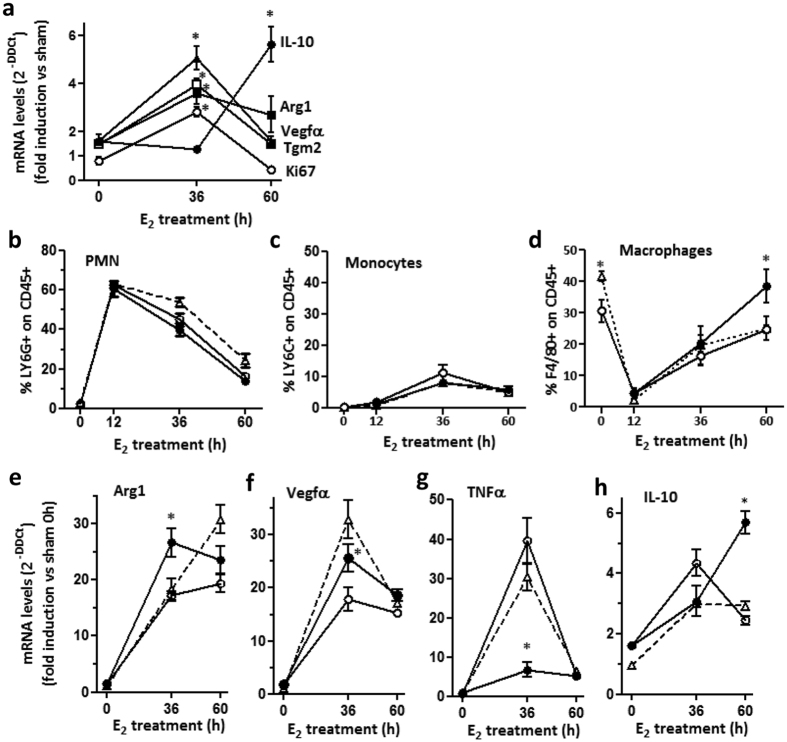
Estrogen induces peritoneal macrophage polarization. (**a**) Peritoneal lavage fluid was collected from sham-operated (not shown), ovariectomized (ovx, shown as time 0) or ovx mice treated with E_2_ for 36 or 60 h. Macrophages were immunosorted and analyzed by real time PCR for gene expression. Data show the expression levels of genes related with proliferation (Ki67, open circles), alternative activation (Arg1, filled squares; Tgm2, open squares) and wound healing (Vegfα, triangles; IL-10, filled circles). Values were calculated using the 2^−ddCt^ method and normalized for the value of the sham group used as reference (=^1^) for each gene. (**b**–**h**) Sham-operated (triangles), ovariectomized (open circles), or ovariectomized and treated with E_2_ (filled circles) animals were injected with zymosan and the number of peritoneal macrophages, polymorphonuclear cells (PMN) and monocytes was analyzed by flow cytometry at different time points (panels b-d, respectively). Immunosorted macrophages were analyzed by real time PCR for the expression of genes, as indicated (panels e–h). Values of the sham group were used as reference (=^1^) and are not shown; ovx values are shown at time 0. Data were calculated using the 2^−ddCt^ method and normalized for the value of the sham group for each gene. Results are expressed as the mean ± SEM (n = 3). *p < 0.05 versus ovx.

Among estrogen-regulated gene ontologies, the *Immune response* and *Wound healing* categories include overlapping functions associated with the alternative polarization of macrophages, a process that progressively shapes macrophage immunometabolic properties to sustain the resolution of inflammation and tissue remodeling. To evaluate macrophage polarization as a dynamic response to estrogen, ovariectomized mice were repeatedly administered estrogen for prolonged periods of time and macrophages were sorted from the peritoneum and analyzed for their expression of genes associated with the polarized activation. Ovariectomy did not alter the basal expression of Arg1, Tgm2, and Vegfα mRNAs, while E_2_ treatment significantly increased the mRNA levels of these M2 markers (Fig. 6a), consistent with our results reported in Fig. 4 and Supplementary Table 1. Of note, also in this setting we detected a significant induction of Ki67 expression, in agreement with the proliferative effect observed on peritoneal macrophages (Fig. 5). Interestingly, polarization gene expression returned to basal levels after 60 h of estrogen replacement, suggesting that this late time point was either associated with a reduced responsiveness to estrogen or that a phenotypic transition of macrophages to the resolution phase was occurring^27^. Consistent with this latter hypothesis, after long time exposure to estrogen macrophages showed a significant increase in IL-10 expression (Fig. 6a), a key mediator in the resolution phase of inflammation^27^, together with a reduction in F4/80 mean fluorescence intensity (see Fig. 5e and Supplementary Fig. 2). These results demonstrate that, beyond proliferation, estrogen induces polarization phenotype in peritoneal macrophages that progressively evolves with time through specialized anti-inflammatory and reparative immune properties.

To define the functional relevance of these events, we adopted a mouse model of transient self-resolving peritoneal inflammation induced by zymosan injection, in which the initial phase of inflammation associated with a massive recruitment of PMN and a concomitant reduction in the number of peritoneal macrophages is followed by the spontaneous resolution of the inflammatory process accompanied by a modest recruitment of monocyte and the progressive return to normal values of peritoneal macrophages^28^,^29^. The role of estrogen in the model was evaluated treating ovx mice with vehicle or E_2_ immediately before zymosan injection and after 24 and 48 h and monitoring its impact on the kinetics of infiltrating cells during the course of inflammation. Sham-operated female mice were used as controls. In agreement with results shown in Fig. 5f, neither ovx nor estrogen administration to ovx mice influenced the number of PMN or monocytes, which varies in the peritoneum during the course of the inflammatory reaction (Fig. 6b and c, respectively). The absence of estrogen did not influence the cellular repopulation of the peritoneum, at least within the time frame analyzed in this experiment, but a significant increase in number of peritoneal macrophages was observed after long term estrogen treatment (Fig. 6d), indicating that estrogen administration provides an immune trigger for macrophages, in agreement with data reported in Fig. 5. Of note, also in these experimental settings estrogen induced the expression of polarization genes in macrophages. Zymosan-induced expression of Arg1 was potentiated by 36 h estrogen treatment in ovx animals, and its reduced expression after 60 h zymosan injection in ovx compared to sham-operated animals is partially rescued by estrogen administration (Fig. 6e). Similarly, the induction of Vegfα mRNA was also modulated by hormone levels after 36 h of inflammation (Fig. 6f). In agreement with this anti-inflammatory effect of estrogen, TNFα expression is increased by hormone loss and strongly reduced by estrogen treatment (Fig. 6g), and IL-10 mRNA levels are significantly higher in animals treated for 60 h with estrogen as compared to sham or ovx animals. Thus, during local inflammation estrogen effects progress with time to support an increase in number of peritoneal macrophages and their alternative polarization to sustain the resolution phase. Altogether, these data demonstrate that estrogen increases macrophage number and dynamically shapes their phenotype to promote their anti-inflammatory and healing properties.

## Discussion

A combination of mechanisms have been shown to support the effects of estrogen on the immune system, including direct genomic and epigenetic effects as well as interactions with transcription factors, co-regulators, cytoplasmic signaling pathways and intercellular communications^4^,^30^,^31^. The present integrated analysis of the *in vivo* response of macrophages to estrogen has revealed the existence of a physiological endocrine-immune crosstalk that links estrogen action with proliferation of peritoneal macrophages and their immunophenotypic adaptation, thus contributing relevant insights to our understanding of estrogen impacts on the regulation of the inflammatory response. The experimental conditions and transcriptomic approach adopted have led to a detailed temporal prospective of this endocrine-immune interplay and have identified a comprehensive list of molecular mediators involved. Clustering analyses according to the onset and duration of estrogen responsiveness clearly showed the progressive response of macrophages to the estrogen signal, as some genes were regulated as either early or late responses to hormone administration, while others resulted in a sustained or potentiated regulation. The biological pathways most prominently modulated included macrophage proliferation, immune response, transcription factor expression, amino acid utilization, and energy metabolism, and on a whole indicated a key role for estrogen as a physiologic immunoregulatory factor for macrophage biology. Though the identification of the molecular mechanisms underlining the genomic events here reported require future investigation, our study reveals the identity and temporal responsiveness of a comprehensive set of novel estrogen-responsive genes (Supplementary Tables 1 and 2) that represent useful biological indicators of hormone signaling in macrophages.

It has recently been demonstrated that peritoneal macrophages are maintained by local self-renewal, with a low level of proliferation throughout life regulated by growth factors and immune mediators including CSF-1 and IL-4^23^,^32^,^33^,^34^,^35^. Functional annotation of DEGs suggested that estrogen also acts as a proliferative signal for peritoneal macrophages. From a physiological point of view, our results provide a novel view of estrogen action in peritoneal homeostasis, in which macrophages are stimulated by estrogen to adapt and develop specific immune regulatory functions, possibly to cope with estrogen-induced changes in the environment. The nature of these concomitant events may include ovulation, with the need of increased macrophage number and activation in order to handle the wound healing response associated with follicle rupture and the induction of an immunotolerant environment which either favors egg fertilization and further implantation or participates in elimination of dead cells and tissue debris resulting from luteal or atretic follicle degradation. The advantage for estrogen to induce macrophage proliferation instead of monocytes recruitment is unclear. As the estrogen surge occurs every 4–5 days in mice and it is perpetuated throughout the fertile life of females, it is possible that such circumstances direct the request of resident macrophages towards the expansion of local cells, a process that is energetically more favorable than the constant increase of hematopoietic precursors and their migration into the peritoneum^35^. This leads to speculate that LPM are the cellular target among peritoneal macrophages that respond to the hormonal signal, although our data only show a trend in the abundance of this macrophage subset following hormone manipulations, without reaching statistical significance (Supplementary Fig. 2). Further studies are thus needed to better characterize the macrophage subset of estrogenic responders. Interestingly, our data also show that genes related with lipid metabolism are modulated by the hormone. Although not addressed in the present study, this evidence represents an additional indication of the metabolic adaptation of macrophages induced by estrogen which might serve the role of directing cholesterol and phospholipid metabolism towards the formation of membranes required for cell proliferation.

Macrophage proliferation has been observed in selected murine and human tumors^36^,^37^, including peritoneal neoplasia^38^,^39^ and in Th2 inflammatory conditions^26^, with variable involvement of IL-4 and CSF-1 depending on the specific experimental conditions^40^. Though a deep investigation of the underlying molecular mechanisms was precluded as estrogen proven to be unable to support macrophage proliferation *in vitro*, similar to what observed for IL-4, in our experimental conditions a significant induction of CSF-1 or IL-4 was not observed, arguing against the hypothesis that the estrogen effect is mediated by these mediators. Indirect mechanisms involving other peritoneal cells able to respond to the hormone cannot be excluded^30^,^41^, however we observed that EREs are present in the promoter of several cell cycle-related genes identified in the present study, including Chaf1a, CcnB2 and Wee1^16^, suggesting a direct binding of estrogen to the promoter of proliferation-related genes in macrophages.

This study also provides the first demonstration that *in vivo* estrogen induces a phenotype resembling alternative macrophage activation that further converts towards a pro-resolving phenotype, as shown by changes induced by hormone replacement on polarization gene expression culminating with the induction of a key immunosuppressive cytokine typically expressed by macrophages during the resolution phase of inflammation, namely IL-10. Importantly, results obtained in the zymosan peritonitis model reveal that these mechanisms are maintained during an acute inflammation of the peritoneum. These results are in agreement with previous studies which suggested that estrogen is involved in macrophage activation and polarization in experimental models of inflammation^42^,^43^,^44^ or in isolated macrophages/cell lines challenged with specific immune stimuli^9^,^15^,^45^. Our study provides a major advancement to this knowledge as it proves estrogen to be *per se* a physiological regulator of the number and reactivity of peritoneal macrophages in intact healthy animals, in the absence of confounding factors such as recruited circulating monocytes achieved in wound healing or in different models of peritonitis.

An abundant number of peritoneal macrophages showing a dysregulated polarization have also been involved in endometriosis, a pathological condition caused by the ectopic growth of endometrial cells in the peritoneum^46^,^47^,^48^. In particular, estrogen is known to drive detrimental effects on endometrial lesion development and progression through its well-recognized activity on endometrial cells^49^,^50^. Macrophages are involved in growth, sustainment and vascularization of endometriosis lesions, and their ability to switch from inflammatory to anti-inflammatory and resolving macrophages is altered in endometriosis^46^,^51^,^52^,^53^. In this respect, the present description of the estrogen-macrophage signaling has intriguing implications for our understanding of endometriosis pathogenesis and therapeutic approaches, and candidate macrophage estrogen-responsive genes identified in this study as potential biomarkers in this clinical setting. Furthermore, the expression of the progesterone receptor is either absent or very limited in peritoneal macrophages, being below the detection limits in our assay. On the contrary, the progesterone receptor is highly expressed in endometrial cells and used as target of current therapeutic interventions for endometriosis that use progesterone analogues to block ER activity. The lack of progesterone receptor in peritoneal macrophages leaves estrogen action unopposed by progestin drugs in these immune cells, with possible reduction in therapeutic efficacy. Although the mechanisms provided in the present work suggest detrimental effect on certain pathologies, such as endometriosis, these events might be involved in the powerful suppressive influence exerted by estrogen in other inflammatory pathologies, such as those affecting the CNS or the lung^8^,^54^.

To conclude, these results represent a key advancement for the understanding of the impact of estrogen on macrophage physiology and identify novel mechanisms and underlying molecular players of potential pathophysiological relevance.

## Methods

### Animal models and treatments.

C57BL/6 female mice at 4 months of age were supplied by Charles River Laboratories; ERαKO female mice were obtained from P. Chambon, IGBMC, Strasbourg, France^55^. Animals were allowed to food and water access *ad libitum* and kept in temperature-controlled facilities on a 12-hour light and dark cycle. Animals were housed in the animal care facility of the Department of Pharmacological and Biomolecular Sciences at the University of Milan. The phase of the reproductive cycle in female mice was assessed by blind analysis of vaginal smears mounted on glass microscope slides and stained with May-Grünwald-Giemsa method (MGG Quick Stain Kit; Bio-Optica) according to the manufacturer’s protocol. 17β-estradiol (E_2_; Sigma-Aldrich) was administered by a 100 μL *s.c.* injection of 5 μg/kg E_2_ dissolved in corn oil by o/n stirring in the dark at room temperature in order to obtain physiologic plasma levels of E_2_^56^. Ovariectomy (ovx) or sham surgery was performed under mild anesthesia obtained by *s.c.* injection of 50 μl solution of ketamine (93.6 mg/kg, Ketavet 100; Intervet) and xylazine (7.2 mg/kg, Rompun; Bayer); control animals received corn oil injection. At the specified time points, animals were euthanized by intraperitoneal (*i.p.*) injection of lethal ketamine and xylazine solution (150 and 12 mg/kg, respectively). Animal groups (n = 3) for the RNA sequencing experiment were given an estrogen-free diet (AIN93M; Mucedola) 2 weeks before and throughout the experiment. For the zymosan peritonitis model, 4 weeks after sham/ovx surgery mice were injected *i.p.* with 1 mg of Zymosan (Sigma-Aldrich) in 1 ml of 0.9% NaCl, while control mice received physiological solution alone. Animals were sacrificed after 12, 36 and 60 h. Ovx mice were treated with corn oil or E_2_ by repeated injections given 3 h before zymosan and 16 h before sacrifice at 36 and 60 h. Macroscopic and weight analyses of uterine tissue were used to confirm the success of ovx and E_2_ treatments. For *in vivo* BrdU labelling experiments, mice were injected i.p. with 30 ul of a 10 mg/ml solution of BrdU (Sigma-Aldrich) dissolved in 0.9% NaCl. Animals were sacrificed 2 h after BrdU injection (n = 4–6). Animal investigation has been conducted in accordance with the ethical standards and according to the Declaration of Helsinki and according to the Guide for the Care and Use of Laboratory Animals, as adopted and promulgated by the US National Institute of Health, and in accordance with the European Guidelines for Animal Care and Use of Experimental Animals. Experiments were approved by the Italian Ministry of Research and University and controlled by an internal panel of experts.

### Peritoneal and bone-marrow-derived macrophages (BMDMs)

Peritoneal cells were recovered by peritoneal lavage. Briefly, 5 ml of pre-chilled 0.9% NaCl were injected into the peritoneal cavity using a 21 G needle, cell suspension centrifuged and resuspended in PBS + 0.5%BSA. For *in vitro* assays, peritoneal cells were incubated with ACK solution (0.15 M NH_4_Cl, 1 mM KHCO_3_, 0.1 mM EDTA; pH 7.3) for 5 minutes at 4 °C and seeded at the concentration of 1 × 10^6^ cells/mL in RPMI (Life Technology-Invitrogen) supplemented with 10% endotoxin-free FBS, 1% penicillin/streptomycin and 1% Na pyruvate. After 45 minutes and several washes in PBS, medium was replaced with RPMI w/o phenol red supplemented with 10% dextran coated charcoal (DCC)-FBS (RPMI + 10% DCC). On the next day, cells were treated for 3 h with vehicle or E_2_ 10^5^ M. For RNA sequencing, gene expression validations and proliferative index analyses peritoneal cells were isolated by anti-CD11b antibody loaded MicroBeads (Miltenyi).; Briefly, 10^7^ peritoneal cells were resuspended in 90 μL PBS + 0.5% BSA, and 10 μL CD11b MicroBeads were added to the cell suspension and incubated for 15 min at 4 °C. After washing, cells were resuspended in 500 uL PBS + 0.5% BSA and applied to LS Miltenyi columns (Miltenyi) for the magnetic separation procedure. After 3 washing steps, CD11b-positive cells were eluted from the columns and counted. Cells were either stored in TRIzol reagent (Invitrogen) for gene expression studies or fixed for flow cytometry analyses. For cell phenotyping and sorting macrophages were analyzed by flow cytometry, as described below.

To prepare BMDMs, bone marrow from the tibia and femur was flushed with RPMI (Life Technology-Invitrogen) using a 21 gauge needle. Cells were centrifuged at 1200 rpm for 5 min at 10 °C, seeded in flask cell culture T75 in DMEM + GlutaMAX (Life Technology-Invitrogen) supplemented with 10% endotoxin-free FBS, 1% penicillin/streptomycin and 1% Na pyruvate and incubated o/n. On the next day, the supernatant was collected, seeded at the concentration of 5–8 × 10^6^ cells/dish and grown for 6 days in DMEM + GlutaMAX containing 20% endotoxin-free FBS, 30% L929-cell conditioned media, 1% penicillin and streptomycin, and 1% Na pyruvate. After 6 days BMDMs were harvested with Accutase (Merck-Millipore) and plated at the concentration of 5 × 10^5^ cells/ml. On the next day, RPMI medium w/o phenol red with 10% dextran coated charcoal-FBS was added and cells were treated for 24 h with vehicle or E_2_ at the specified concentrations; for the 3 h treatment vehicle or E_2_ were added in the last 3 h of incubation.

### RNA preparation and analysis

Total RNA was purified using RNeasy minikit protocol (Qiagen), according to the manufacturer’s instructions, including a step with deoxyribonuclease incubation. For real time PCR, 1 μg RNA (250 ng in peritonitis model) was used for cDNA preparation using 8 U/μl of Moloney murine leukemia virus reverse transcriptase (Promega) in a final volume of 25 μl; the reaction was performed at 37 °C for 1 h, and the enzyme inactivated at 75 °C for 5 min. Control reactions without the addition of the reverse transcription enzyme were performed (data not shown). A 1:4 cDNA dilution was amplified using GoTaq^®^qPCR Master Mix technology (Promega) according to the manufacturer’s protocol. The PCR was carried out in triplicate on a 96-well plate using 7900HT fast real time PCR system (Applied Biosystems) with the following thermal profile: 2 min at 95 °C; 40 cycles, 15 sec at 95 °C, 1 min at 60 °C. Primer sequences are reported in Supplementary Table 3. Data were analyzed using the 2^−ΔΔCt^ method. For RNA sequencing, RNA Quality Control was performed on all RNA samples with an electrophoretic run on a Bioanalyzer instrument using the RNA 6000 Nano Kit (Agilent). RNA Integrity Number was determined for every sample and all the samples were considered suitable for processing based on the RNA integrity (RIN > 8). RNA concentration was estimated through spectrophotometric measurement using a Nanoquant Infinite M200 instrument (Tecan). Sequencing libraries were prepared using the TruSeq™ RNA Sample Preparation Kit (Illumina) using 1.8 ug of total RNA as input. Polyadenylated transcripts were purified using poly-T oligo-attached magnetic beads. PolyA RNA was fragmented at 94 °C for 8 min and retrotranscribed using random hexamers. Multiple indexing adapters were ligated to the ends of the cDNA and the amount of DNA in the library was amplified with 10 PCR cycles. Final libraries were validated and quantified with the DNA1000 kit on the Agilent Bioanalyzer Instrument. Pooled libraries were sequenced on the Illumina Genome Analyzer II_x_ producing an average of 13 M reads per library.

### Bioinformatics analysis

BaseCall files were converted to FastQ files using Casava 1.8.2. Sequencing reads were aligned to the mouse genome (mm10) using TopHat v.2.0.9. Transcripts were reconstructed and quantified using Cufflinks v2.1.1 and differential expression analysis was performed using CuffDiff^58^. CuffDiff uses the test statistics T = E[log(y)]/Var[log(y)], where y is the ratio of the normalized counts between two conditions. A t-test is used to calculate the P value for Differential Expression^59^. A threshold of 0.05 was applied to False Discovery Rate (FDR) adjusted p values in order to select the differentially expressed genes (DEGs) to use in downstream analysis; we also included genes with a log2 fold-change (lgFC) > 1 either showing one anomalous triplicate FPKM value and an FPKM average value between 1 and 2. Heat map of DEGs was made with Genesis software using triplicates mean, after normalization and log2 transformation. Cluster analysis was performed with the Genesis software tool using k-means clustering function (k = 8) in order to identify group of genes with a similar regulation at 3 and 24 h of treatment^60^; genes with lgFC > +/−0.40 were selected. In each cluster of genes, the regulatory sequences in 20 Kb around the transcription start site were analyzed using iRegulon Cytoscape App and candidate transcription factors were predicted^61^. Overrepresentation analysis (ORA) on DEGs lists was performed using the Functional Annotation Tool in DAVID website^62^. The lists of DEGs at 3 and 24 h of estradiol treatment were used as input gene list and the mouse genome was used as background list. Biological processes, molecular functions and KEGG pathways were investigated focusing on enriched terms with a Benjamini adjusted p-value less than 0.05. A Protein-Protein Interaction Network of the differentially expressed genes has been created using STRING^63^.

### Flow cytometry analysis

To assess cell proliferation index, CD11b-positive cells obtained from each animal were divided in 3 aliquots; 2 aliquots of 2.5 × 10^5^ cells each were used to detect BrdU and Ki67 by flow cytometry, the remaining aliquot was used for gene expression. Cells were suspended in 2 ml of pre-chilled ACK solution. For Ki67 staining, cells were fixed in 4% paraformaldehyde for 15 min, extensively washed with 125 mM glycine in PBS and permeabilized o/n in PBS containing 0,5% Triton X-100 and 1% BSA, at 4 °C. Cells were incubated with rabbit anti-mouse Ki67 antibody conjugated with eFluor660 (eBioscence-Affymetrix) diluted 1:100 in Incubation Solution (PBS containing 0.5% Triton X-100 and 0.05% BSA) at room temperature for 1 h. After extensive washes in PBS, cells were analyzed with a flow cytometry system (BD FACS Calibur). For BrdU staining, cells were fixed and permeabilized in 70% EtOH for 30 min at 4 °C and DNA was denaturated with 2 N HCl/0.5% Triton X-100 and incubated 30 min at room temperature. Cells were washed with 0.1 M sodium tetraborate (pH 8.5) and incubated with rat anti-mouse BrdU antibody (AbD Serotec) diluted 1:100 in Incubation Solution (PBS containing 0.05% Tween-20 and 1% BSA). After washes in PBS/1%BSA, cells were incubated with Alexa488-conjugated goat anti-rat secondary antibody (1:200 in incubation solution; Molecular Probes) for 1 h at room temperature. Cells were extensively washed with PBS, resuspended in PI solution (H_2_O containing 10% NP40, 1 mg/ml RNase A and 5 μg/ml PI stock; Sigma-Aldrich). Samples were analyzed using FACSCalibur and analyzed with FlowJo version 9 software (Tree Star). Animals with no pulse of BrdU were used for gating strategy to evaluate nonspecific signals.

In the sham/ovx animals and in the zymosan-induced peritonitis model, immunofluorescent staining of peritoneal cells was performed with V450 anti-mouse CD45, PerCP-Cy5.5 anti- mouse CD11b, FITC anti-mouse Ly6C, PE-Cy7 anti-mouse Ly6G, PE anti-mouse CD11c, PE anti-mouse CD19 (BD Biosciences), AlexaFluor 647 anti-mouse F4/80 (AbD Serotec) and PE anti-mouse CCR3 (R&D Systems). Samples were analyzed using FACS Canto II and DIVA software (BD Biosciences); gating strategies are reported in Supplementary Fig. 1. Resolution indices of zymosan-induced peritonitis were calculated as previously described^57^. Ly6G-negative CD11b-positive and F4/80-positive macrophages were sorted using sorter Aria (FACS Aria, Becton Dickinson Biosciences) and resuspended in TRIzol reagent for gene expression analyses.

### Data availability

Data is available at the NCBI repository through the link http://www.ncbi.nlm.nih.gov/bioproject/376257.

### Statistical analysis

Statistical significance was carried out with the GraphPad Prism version 5.02 for Windows by a Bonferroni *post hoc* test for multiple comparisons. Unless otherwise stated, “n” indicates biological replicates.

## Additional Information

**How to cite this article**: Pepe, G. *et al*. Self-renewal and phenotypic conversion are the main physiological responses of macrophages to the endogenous estrogen surge. *Sci. Rep.* **7**, 44270; doi: 10.1038/srep44270 (2017).

**Publisher's note:** Springer Nature remains neutral with regard to jurisdictional claims in published maps and institutional affiliations.

## Supplementary Material

Supplementary Information
