# Supplementary material for: Self-renewal and phenotypic conversion are the main physiological responses of macrophages to the endogenous estrogen surge
Source: Sci Rep. 2017 Mar 20;7:44270. doi: 10.1038/srep44270 (PMC5357836; doi:10.1038/srep44270)
Supplement: Supplementary Information [file srep44270-s1.pdf]

Self-renewal and phenotypic conversion are the main physiological responses of macrophages to the endogenous estrogen surge

Giovanna Pepe[1], Daniele Braga[2,3], Tiziana A. Renzi[4,5], Alessandro Villa[1], Chiara Bolego[6], Francesca D'Avila[2,3], Cristina Barlassina[2,3], Adriana Maggi[1], Massimo Locati[4,5] and Elisabetta Vegeto[1*]

Supplementary Table 1. **List of E_2-regulated genes in macrophages**.

Differentially regulated genes (DEGs) in macrophages following 3 and 24 h of estrogen administration to metaestrous female mice are listed according to logFC; cluster and ontologies associations and the number of ERE in gene promoter/enhancer are reported.

GENE	logFC		Cluster	ERE	Ontologies
	3h	24h			
Vegfa*	3.22		I	1	IR; WH; TX; AP
H2-M3	1.97		I		IR
Lbx2§	1.80		I		TX
Rpl39l*	1.56		I		
Hgd	1.53		I		
Fam187b*	1.47		I		
Prelid2*	1.43		I		
Ubxn6*	1.41		I		
Gsg1*	1.40		I		
Cdk1	1.39		I		CC; SR
Ofcc1*	1.32		I		
Rad51ap1§	1.27		I		SR
Ckap2l	1.22		I		
Kif22	1.18		I		SR
Oaf*	1.17		I		
Mxd3*	1.16		I		TX
Itga7*	1.15		I		
Spc25*	1.15		I		CC
Cd46*	1.13		I		
Trappc1§	1.13		I		
Egr3	1.12		I		TX
Cenph*	1.09		I		CC
Depdc1b*	1.08		I		
Il1b*	1.08		I		IR; WH; LM
Plekhf1*	1.07		I		AP
Chaf1b*	1.07		I		CC; TX; SR
Cdca5§	1.06		I		CC
Pole*	1.04		I	1	SR
Lpl	1.03		I	3	
Tgfbr3	1.03		I		WH; TX
Fam101b§	1.01		I		
Cdca7	0.99		I		TX
Sox7	0.97		I	1	TX
Gmnn	0.95		I		CC
Plod3	0.95		I		
Asf1b	0.90		I		TX
Cxcr7	0.83		I		
Lonrf3	0.83		I		
Loxl2	0.81		I		
Nlrp3	0.79		I		IR; WH; TX; AP
Tipin	0.76		I		SR; CC
Nr4a1	0.76		I	1	TX; AP
Hes1	0.71		I		TX
Tacc3	0.70		I		CC; WH
Itgb3	0.69	-0.63	I		
Fabp7	0.67		I	1	
Slpi	0.65		I	1	
Cdkn1a	0.63		I	1	CC; AP; SR
Id2	0.63		I		WH; TX
Klf4	0.62		I		TX
Rab27a	0.52		I		IR; WH; AP
Lrrk2	0.51		I		
Cdc42ep4	0.47		I	1	
Ccr2	0.44		I		IR; WH
Fhit*	-2.17		I		
Ccl5	-2.14		I		IR; WH
Ms4a4b	-2.12		I		
Il7r*	-1.89		I	1	IR; WH
Cldn20§	-1.59		I		
Lat*	-1.56		I		IR; WH
Il2rb	-1.45		I	2	AP
Slc18a2§	-1.43		I		
Mylpf*	-1.42		I	2	
Gm20735*	-1.36		I		
Serpine1*	-1.27		I	1	
Dnajb13§	-1.24		I		PF
Oas1g*	-1.24		I		IR
Gm14393§	-1.17		I		
Dhrs13*	-1.16		I		
Sec16b	-1.15		I		
Bri3*	-1.13		I		
Per1	-1.13		I	1	TX
Ifitm1§	-1.10		I		
Tcf7	-1.09		I	1	TX; AP
Ly6c, Ly6i*	-1.09		I	1	
Lck*	-1.04		I		
Tmem220*	-1.02		I		
Cyp11a1§	-1.01		I		
Lyve1	-0.81		I		
Usp18	-0.80		I		
Dnajb1	-0.68		I		PF
Per3	-0.63		I		TX
Tbc1d9	-0.61		I		
Ccr7	-0.57		I		IR
Frmd4b	-0.57		I		
Ltb	-0.51		I	1	IR; WH; AP
Pou2f1	-0.49		I		TX
Manf	-0.47		I		
March1	-0.44		I		
Chaf1a**	1.55	1.34	II	1	CC; TX; SR
S100a5§,**	1.37	1.45	II	1	
Zfp580*,**	1.32	1.03	II		TX
Bub1b	1.3	1.38	II		CC
Prc1	1.22	1.23	II		CC
Cx3cr1§,**	1.20	1.13	II	1	IR; AP
Ccr6	1.16	1.29	II		
Cenpk*,**	1.14	1.20	II		TX
Nusap1	1.08	1.19	II		CC
Serpinb2	1.03	1.00	II		
Ube2c*,**	1.00	1.07	II		CC
Fam217b	0.95	0.95	II		
Slc16a3	0.92	0.59	II		
Ldlr	0.82	0.47	II		IR; LM
Mcm5	0.82	0.78	II		TX
Uhrf1	0.77	0.81	II		CC; TX; SR
Lig1	0.76	0.83	II		CC; SR
Ptgir	0.74	0.50	II		
Hk2	0.72	0.60	II		
Incenp	0.67	0.60	II	2	CC
Mapk6	0.64	0.51	II		CC
Adam8	0.63	0.61	II		
E2f3	0.60	<0.4	II		CC; TX
Bcl3	0.60	<0.4	II		IR; WH; TX; AP; SR
Fam171a1	0.59	<0.4	II		
Slc9a9	0.59	<0.4	II		
Cd40	0.58	<0.4	II		
Cdkn2d	0.57	<0.4	II		CC
Arf2	0.57	0.45	II		
Dram1	0.57	<0.4	II		
Ripk3	0.56	0.57	II		AP
Kremen1	0.54	<0.4	II		
Abcg1	0.54	<0.4	II	1	LM; TX
Padi4	0.53	<0.4	II	1	TX
5430435G22Rik/Rab7b	0.51	<0.4	II		
Arhgef10l	0.50	<0.4	II		
Steap3	0.49	0.57	II		CC
Plscr1	0.46	<0.4	II	1	WH
B430306N03Rik/Treml6	0.45	0.46	II	2	
Dmpk	0.44	<0.4	II		
Mcm2	<0.4	0.53	II		CC
Mrgprb2*,**	-5.04	-2.44	II		
Cpa3*,**,^	-4.57	-1.90	II		
Mcpt4*,**,^	-3.91	-1.65	II		
Cma1*,**,^	-3.80	-1.87	II		
Tpsb2*,**,^	-3.72	-1.83	II		
Mrgprb1*,**	-3.57	-1.21	II		
Kit*,**,^	-2.90	-1.43	II	2	
Angptl4	-2.76	-1.51	II	2	LM; AP; SR
Slc6a4*,**	-2.50	-2.32	II	1	
Pdk4	-2.13	-2.46	II	2	
Ly6k	-1.86	-1.74	II		
Cxcr4	-1.32	-0.83	II		WH
Hsph1	-1.29	-1.28	II		PF
Wee1	-1.23	-1.13	II	2	CC
Dgke	-1.10	-0.74	II		
Mbd1	-1.04	-1.09	II	1	TX
Jdp2	-0.96	-0.48°	II		TX
Klf9	-0.92	-0.65	II		TX
Fam46c	-0.91	-0.74°	II		
Gas5,Mir5117	-0.90	-0.95	II		
Per2	-0.85	-0.81	II	2	TX
Stip1	-0.77	-0.58	II	1	
Cpt1a	-0.77	-0.56	II		
Cep85	-0.77	-0.91	II		
Pik3r5	-0.76	-0.79	II		
Hsp90aa1	-0.74	<0.4	II		PF

Ddit4	-0.74	-0.62	II		
Pgm2l1	-0.73	-0.55	II		
Herpud1	-0.72	-0.56	II	1	
Trim65	-0.70	<0.4	II		
Sfn	-0.69	<0.4	II		
Sik1	-0.68	-0.54	II		CC
Dnaja1	-0.68	-0.62	II	1	PF
Cacna1e	-0.68	<0.4	II		
Mll1	-0.67	-0.60	II		WH; TX
Tagap	-0.67	-0.56	II		
Oas2	-0.66	<0.4	II		IR
Trim47	-0.66	-0.73	II		
Stxbp3a	-0.66	-0.71	II		
Trim7	-0.63	-0.56°	II		
Nr1d2	-0.62	<0.4	II		TX
Erdr1	-0.60	-0.50	II		
Tef	-0.60	<0.4	II		TX
Ino80d	-0.60	-0.54	II		
Rusc2	-0.59	-0.66	II		
Slc37a2	-0.59	-0.54	II		
Jmy	-0.58	<0.4	II		CC; TX; AP; SR
Rnf169	-0.57	<0.4	II		
Fkbp4	-0.56	<0.4	II	1	
Tsc22d3	-0.56	<0.4	II		TX; AP
Atxn1	-0.56	<0.4	II		TX
Lmo7	-0.51	<0.4	II		
Tfb2m	-0.51	<0.4	II		TX
Rabgap1l	-0.51	<0.4	II		
Ctns	-0.51	-0.53	II	1	
Phf15	-0.50	<0.4	II		
Epas1	-0.50	-0.56	II	1	WH; TX; SR
Mlxip	-0.50	-0.44	II		TX
Slc16a10	-0.48	<0.4	II		
Ypel2	-0.47	-0.49	II		
Ptpn22	-0.46	<0.4	II		WH
Cacybp	-0.45	<0.4	II	1	
Ubfd1	-0.44	<0.4	II		
Glcci1	-0.44	-0.5	II	1	
Il16	-0.43	<0.4	II	1	IR; TX
Rc3h1	-0.43	<0.4	II		
Ahsa1	-0.43	<0.4	II		PF
Crebbp	<0.4	-0.5	II		
Diras2	<0.4	-0.41	II		
Bex6**		4.46	III		
Spp1*,**	1.51	3.96	III		AP
Arg1	0.81	2.81	III		WH
Trem1*	1.72	2.09	III	2	
Nuf2*,**	1.41	1.86	III		CC
Birc5*	1.26	1.78	III		CC; AP
Ccnb2	1.38	1.76	III	1	CC; WH
Kifc1		1.72	III		CC
Gins2§	1.34	1.71	III		
Spc24§	1.09	1.70	III		CC
Fignl1*	1.18	1.47	III	1	
Ccnb1		1.45	III		CC
Ccnf		1.45	III		CC
Top2a	1.14	1.43	III	1	TX
Mcm10*,**	1.07	1.41	III		
Camkk1		1.40	III	1	
Gm16907**		1.39	III		
Cdca3		1.36	III		CC
Mag§§		1.35	III		
Kif11		1.29	III		CC
Rn45s	0.59	1.26	III		
Slc35g2§§		1.25	III		
Mki67	0.94	1.24	III		CC
Stmn1	0.89	1.24	III		CC
Timeless**		1.19	III	1	CC
Tpx2		1.16	III		CC
Fxn§§		1.15	III		
Slc7a8	0.85	1.13	III		
Smim5§§		1.09	III		
Gm10814**		1.08	III		
Dtl**		1.07	III		
Mefv§§		1.07	III		WH
Cdc6*		1.04	III		CC
Ccr5	0.5	1.02	III		IR; WH
Kif23		1.02	III		
Syce2**		1.01	III		CC
Echdc3**		1.01	III		
Lair1	0.54	0.99	III		
Dnajb5		0.98	III	1	
Rrm2		0.95	III	2	
Tgm2		0.94	III	1	WH
Slfn9		0.94	III		
Marco	0.46	0.93	III	1	
Fcgr4	0.45	0.91	III		
Tgm1		0.91	III	1	
Aurkb		0.86	III		CC
Arhgap19		0.86	III		
Emp1		0.86	III		
Layn		0.83	III		
Idi1		0.82	III		LM
Guca1a		0.79	III	1	
Stxbp1		0.78	III	1	IR
Cd300lf		0.78	III		IR
Dab2		0.77	III	1	IR
Il21r		0.77	III		
Ckb		0.76	III		
Cwc27		0.74	III		
Gm6682		0.74	III		
Dusp16		0.73	III		
Arl4c		0.70	III		
Srxn1		0.67	III		
Dhcr24		0.65	III	2	LM
Sel1l3		0.65	III		
Kpna2		0.64	III	1	
Kank2	0.51	0.64	III		
Msr1		0.62	III		IR; LM
Fcrl5		0.62	III		
Pgk1		0.61	III		
Fmnl3		0.60	III		
Dpysl3		0.60	III		
Pkm		0.60	III		
Lbp		0.60	III		IR; WH; LM
Cd300ld		0.59	III		IR
Jam2		0.59	III		
Pla2g4a		0.58	III	1	
Fus		0.57	III		
Fgr		0.57	III		
C1ra		0.57	III		IR; WH
Cd22		0.56	III	1	
Phyh		0.56	III		
Pdk1		0.56	III		
Rfx5		0.54	III		
Rhoh		0.53	III		
Lars		0.53	III		
Tlr1		0.51	III	1	IR; WH
Nin		0.50	III		
Itga9		0.50	III	1	
Ifi30,Pik3r2		0.50	III		
Lrrc25		0.49	III		
Myo1e		0.49	III		WH
Rhoc		0.47	III		
Impdh1		0.46	III		
Tpi1		0.46	III		
Ap2m1		0.46	III	1	
Ldha		0.45	III		
Jarid2		0.44	III		
Serpine2		-2.66	III	1	
Rasl10a*,**	-1.19	-2.55	III		
Gm16701		-2.44	III		
Fkbp5	-1.35	-2.37	III	2	PF
Zbtb16	-1.09	-2.32	III		WH; TX; AP
Hspb1	-1.33	-2.29	III		
Slc15a2		-2.19	III		
Klf15*,**	-1.34	-2.05	III		TX
Tcf23**		-1.91	III		
Zfp811	-0.87	-1.89	III		TX
Rfx2	-0.55	-1.64	III		TX
Tppp	-0.51	-1.61	III		
Cyp26a1	-0.96	-1.58	III		
Plekha6	-0.56	-1.54	III		
Gpr114**		-1.53	III		
Serf1**		-1.50	III	1	
Map3k6	-0.72	-1.49	III	1	
Shank3		-1.46	III	1	
Ralgds	-0.74	-1.45	III		
Lims2		-1.44	III		
Prtg		-1.39	III		
Krt7		-1.36	III		CC
Eps8l1		-1.35	III		
Apoc1	-0.54	-1.29	III		LM
Fam222a**		-1.28	III		
Thbd	-0.6	-1.26	III		WH
Ryr2		-1.24	III		
Tacc2		-1.18	III		CC
Klf11	-0.68	-1.18	III		WH; TX

Fcer1a^		-1.17	III		
Tmem150b**		-1.12	III		
Il6**		-1.10	III		WH
Orm2,Orm3§§		-1.09	III		WH
Plin2	-0.78	-1.05	III		LM
Il1rl1	-0.64	-1.05	III	2	IR
Ccdc15§§		-1.04	III		
Frat2		-1.01	III		
Slc10a6		-0.98	III		
Tnfsf8		-0.93	III		IR
Tnfrsf8		-0.92	III		
Cpm		-0.92	III		
Ier3		-0.91	III		
Cd93	-0.53	-0.90	III		
Ccm2l		-0.89	III		
Banp	-0.64	-0.89	III		CC
Stxbp3b		-0.88	III		
Sgms1	-0.6	-0.84	III		WH; AP
Rai14		-0.84	III		
Glul		-0.80	III		
Hilpda		-0.80	III		
Sult1a1		-0.78	III		
Engase	-0.5	-0.77	III		
Hist1h2bc		-0.76	III		
Nfkbiz		-0.76	III		WH
Zfp97		-0.75	III		
Elovl5		-0.75	III		
Mst1r		-0.73	III	2	
Snta1		-0.73	III	1	
Nrip1		-0.71	III	1	LM
Irf2bp2		-0.71	III		
Zkscan3		-0.69	III		
Mdm1		-0.69	III		
Cdan1		-0.69	III		
Tns1		-0.68	III		
Epha2		-0.68	III	1	WH
Igf1r		-0.68	III		
Dusp6		-0.68	III		
Slc27a1		-0.67	III	1	LM
Prkar2b	-0.49	-0.66	III	1	
Snhg11		-0.66	III		
Chordc1		-0.66	III	1	
Dapk1		-0.64	III		
Dhrs3		-0.63	III		
Wdr45		-0.62	III		
Cd300a		-0.62	III		IR
Sh3bgrl2		-0.61	III		
Ulk1		-0.61	III	1	IR
Notch1		-0.61	III		WH
Edil3		-0.60	III		
Zbtb44		-0.58	III		
Mrvi1		-0.57	III		
Zfp36l1		-0.57	III	1	WH
Cds2		-0.56	III		
Klhl24		-0.56	III		
Cnst		-0.55	III		
Dennd4c		-0.55	III		
Efcab4a,Pnpla2		-0.55	III		
Dnase1l2,E4f1		-0.55	III		CC
Net1		-0.54	III		
2210018M11Rik/Emsy		-0.53	III		
Sorbs3		-0.53	III		
Pcmtd2		-0.53	III		
Ppl		-0.52	III	2	
Hspa12a		-0.51	III		
Arrdc3		-0.51	III		
Zmiz1		-0.51	III		WH
Fam20c		-0.51	III		
Fam46a		-0.50	III		
Mob3b		-0.50	III		
Pgap1		-0.49	III		
Rfwd2		-0.48	III		
Ubn2		-0.48	III		
Bach1		-0.48	III		
Ube2h		-0.48	III		
Jag1		-0.47	III	1	
Wnt2		-0.47	III		WH
Clec10a		-0.47	III		
Pnpla7		-0.46	III		
Dapp1		-0.46	III		
Pan3		-0.46	III		
Rassf3		-0.45	III		
Heca		-0.44	III		
Slc25a37		-0.43	III		
Zscan26		-0.43	III		
Dusp11		-0.42	III		
Ccl12**		4.10	IV		IR; WH
Upp1**		3.30	IV		
Rab3il1		2.31	IV		
Chi3l3		2.16	IV	1	WH
AA467197/Nmes1**		2.05	IV		
Vcan		2.00	IV		
3110057O12Rik/Abhd18		1.95	IV		
Rarres2§§		1.75	IV		
Gas6		1.68	IV	1	
Ckap2**		1.67	IV		CC
Vpreb3**		1.66	IV	2	
Siglec1		1.60	IV		
Aif1		1.55	IV	2	
Ccl22**		1.53	IV		IR; WH
Gm9920§§		1.50	IV		
Ucp1§§		1.40	IV		
Isg15		1.37	IV	1	
Chst11		1.37	IV		
Lars2		1.36	IV		
Rnf128		1.33	IV		
Eme1**		1.32	IV		
Ifit3		1.31	IV		
Nxpe5		1.29	IV		
Il2ra		1.27	IV		
Cmss1		1.23	IV		
Fdps		1.19	IV	2	LM
Fcgr1		1.17	IV		IR; WH
Sfxn5		1.13	IV		
Papss2		1.12	IV		WH
Trem2		1.09	IV		
Ngfr		1.08	IV		WH
Gatm§§		1.04	IV		WH
Rsad2		1.03	IV		IR
Soat2		0.97	IV		LM
Mvb12b		0.95	IV		
Nme4		0.95	IV		
Tmem8		0.95	IV	1	
Fam213b		0.94	IV		
P2ry6		0.94	IV		
Folr2		0.93	IV		
Irf7		0.87	IV		IR
Cbr2		0.86	IV		
Ifi27l2a		0.86	IV		
Slfn1		0.85	IV	1	CC
Cplx2		0.85	IV		IR
Ms4a6c		0.83	IV		
Mlkl		0.82	IV		
Ppapdc1b		0.82	IV		
Adap2		0.80	IV		
Galk1		0.77	IV		
Glrx		0.76	IV		
H2-Eb2		0.76	IV		IR
Marcksl1		0.76	IV		
Zbp1		0.75	IV		
Nrp1		0.74	IV		WH
Fpr2		0.72	IV		IR
Oas3		0.72	IV		IR
Cpne2		0.71	IV		
Pram1		0.70	IV		
Cd163		0.68	IV		WH
Pam		0.68	IV		
Nlrc3		0.67	IV		
Ms4a6d		0.66	IV		
Dhx58		0.64	IV		IR
Ddx60		0.64	IV		
Fcrl1		0.63	IV		
Epb4.1l1		0.62	IV		
Tnfrsf11a		0.61	IV		
Rbm3		0.61	IV		
Dhx29		0.57	IV		
Pik3ap1		0.56	IV		
Rhobtb1		0.55	IV		
Slfn5		0.55	IV		
Ctsc		0.54	IV		
Ckap4		0.54	IV		
Acp2		0.53	IV	2	
Lpin2		0.52	IV		
Plcb1		0.52	IV		
Dck		0.52	IV		
Atrip,Trex1		0.52	IV		
Tpcn2		0.52	IV		
Lgals1		0.51	IV	3	

Lman1		0.49	IV		
Lrrc59		0.49	IV		
F13a1		0.49	IV		WH
Mrc1		0.49	IV		IR
Etv5		0.49	IV	1	
Rtp4		0.48	IV		
Icam1		0.48	IV	1	IR
Cd38		0.46	IV		
Nme1		0.46	IV		IR
Ncbp1		0.45	IV		
Stat1		0.45	IV		
Ostc		0.44	IV		
Rgs1		-2.44	IV		
Mylk3§§		-1.88	IV		
Abca6		-1.84	IV	1	
Spa17**		-1.72	IV		
Hhipl1		-1.71	IV		
Ccl3		-1.66	IV		IR; WH
Nupr1l§§		-1.66	IV		
Dusp1		-1.49	IV		CC
Fos		-1.34	IV		
Xlr3b**		-1.26	IV		
Nr1d1		-1.19	IV	2	
Rab44		-1.15	IV		
Gm15471**		-1.15	IV		
Cxcl2		-1.1	IV	2	IR; WH
Cables1		-1.07	IV		CC
Hgf		-1.03	IV	1	
Gfod1		-1.01	IV		
Art3**		-1.01	IV		
Zfp72**		-1.00	IV		
Egr1		-1.00	IV	1	
Tppp3		-0.99	IV		
Adamtsl4		-0.95	IV		
Fosb		-0.93	IV	1	
Nt5e		-0.91	IV		
Zfp36		-0.87	IV	2	WH
Ccrl2		-0.87	IV		
Tox2		-0.86	IV		
Rgs2		-0.85	IV		CC
Ppap2a		-0.84	IV	1	
Cyp2ab1		-0.84	IV		
Insr		-0.81	IV		
Nedd4		-0.79	IV		
Tmem62		-0.76	IV		
Aqp9		-0.75	IV		
Hr		-0.74	IV	1	
Fabp4		-0.72	IV		
Jun		-0.69	IV		
Garnl3		-0.65	IV		
Ocln		-0.65	IV		
Abca9		-0.64	IV		
Rbpms		-0.62	IV		
Cd14		-0.60	IV	2	IR; WH
Fgfr1		-0.60	IV		WH
Hist1h1c		-0.60	IV		
Slc22a17		-0.60	IV		
Tln2		-0.60	IV		
Cav1		-0.58	IV		IR; WH; LM
Plcb4		-0.58	IV		
Nfia		-0.57	IV		
Egln3		-0.57	IV		
Ston2		-0.56	IV		IR
Tgfb2		-0.53	IV	2	IR; WH
Calcoco1		-0.52	IV		
Myadm		-0.51	IV		
Pi16		-0.51	IV		
Icam2		-0.50	IV	1	
Gata6		-0.50	IV	1	
Csf3r		-0.50	IV		IR
Cask		-0.47	IV	1	
Lama3		-0.46	IV		
Parvb		-0.46	IV		
Fcrls		-0.46	IV		
Slc9a3r2		-0.46	IV		
Junb		-0.45	IV		WH
Mgst1		-0.43	IV		

Significant ontologies are reported as: AP, apoptosis; CC, cell cycle; IR, immune response; LM, lipid metabolism; PF, protein folding; SR, stress response; TX, transcription factors; WH, wound healing.

*DEGs excluding anomalous value at 3h, **DEGs excluding anomalous value at 24h; §DEGs with FPKM <2 and >1 at 3h; §§DEGs with FPKM <2 and >1 at 24h; ^genes excluded from GO analysis; °not a DEG value.

Supplementary Table 2. Estrogen-regulated biological pathways in macrophages. Functional annotation analysis on the differentially expressed genes (DEGs) lists from 3 and 24 h estrogen treatment was performed using Gene Ontology.

Ontologies		DEGs at 3h	DEGs at 24h	Grouped categories
ID	Name			
GO:0007049	Cell cycle	**CHAF1A, NUF2, CDK1, CCNB2, BUB1B, BIRC5, PRC1, SPC25, SPC24, CENPH, NUSAP1, CHAF1B, CDCA5, UBE2C, GMNN, MKI67, STMN1, UHRF1, TIPIN, LIG1, TACC3, INCENP, MAPK6, CDKN1A, E2F3, CDKN2D, STEAP3**, WEE1, SIK1, BANP, JMY	**NUF2, BIRC5, CCNB2, KIFC1, SPC24, CKAP2, CCNF, CCNB1, BUB1B, CDCA3, CHAF1A, KIF11, MKI67, STMN1, PRC1, NUSAP1, TIMELESS, TPX2, UBE2C, CDC6, SYCE2, AURKB, UHRF1, SLFN1, LIG1, INCENP, STEAP3, MCM2, MAPK6**, DUSP1, KRT7, TACC2, WEE1, CABLES1, BANP, RGS2, E4F1, SIK1	Cell cycle (CC)
GO:0006955	Immune response	**VEGFA, H2-M3, CX3CR1, IL1B, NLRP3, BCL3, RAB27A, CCR5, CCR2**, CCL5, IL7R, LAT, OAS1G, OAS2, IL1RL1, CCR7, LTB	**CCL12, CCL22, FCGR1, CX3CR1, RSAD2, CCR5, IRF7, CPLX2, CD300LF, H2-EB2, OAS3, DHX58, LBP, CD300LD, C1RA, TLR1, ICAM1**, CCL3, IL1RL1, CXCL2, IL16, TNFSF8, CD300A, CD14	Immune response (IR)
GO:0006935	Chemotaxis		**CCL12, CCL22, FPR2, LBP**, CCL3, CXCL2, TGFB2, CX3CR1, CSF3R	
GO:0006897	Endocytosis		**FCGR1, STXBP1, DAB2, MSR1, LBP, MRC1, LDLR, NME1**, ULK1, CAV1, STON2	
GO:0009611	Response to wounding	**IL1B, ARG1, NLRP3, RAB27A, CCR5, CCR2**, CCL5, LAT, SGMS1, THBD	**CCL12, ARG1, CHI3L3, CCL22, FCGR1, , PAPSS2, MEFV, GATM, CCR5, NGFR, CD163, LBP, C1RA,TLR1, F13A1**, CCL3, THBD, IL6, CXCL2, ORM2, ORM3, SGMS1, NFKBIZ, CD14, TGFB2	Wound healing (WH)
GO:0048534	Hemopoietic/ lymphoid organ development	**VEGFA, CCNB2, TGFBR3, TACC3, ID2, BCL3, PLSCR1, CCR2**, IL7R, ZBTB16, KLF11, MLL1, LTB, EPAS1, PTPN22		
GO:0001568	Blood vessel development		**TGM2, NRP1, MYO1E**, CXCR4, EPHA2, FGFR1, NOTCH1, CAV1, EPAS1, ZFP36L1, ZMIZ1, TGFB2, WNT2, JUNB	
GO:0045834	Positive regulation of lipid metabolic process	**IL1B, ABCG1**, ANGPTL4		Lipid metabolism (LM)
GO:0008203	Cholesterol metabolic process		**FDPS, SOAT2, IDI1, LDLR**, APOC1, DHCR24	
GO:0010876	Lipid localization		**MSR1, LBP, LDLR**, APOC1, PLIN2, SLC27A1, CAV1, NRIP1	
GO:0006355	Regulation of transcription	**VEGFA, LBX2, CHAF1A, ZFP580, MXD3, CENPK, TOP2A, EGR3, CHAF1B, TGFBR3, CDCA7, SOX7, ASF1B, MCM5, NLRP3, UHRF1, NR4A1, HES1, ID2, KLF4, E2F3, BCL3, ABCG1, PADI4**, KLF15, PER1, ZBTB16, TCF7, MBD1, JDP2, KLF9, ZFP811, PER2, KLF11, MLL1, PER3, NR1D2, TEF, JMY, TFB2M, TSC22D3, ATXN1, RFX2, EPAS1, MLXIP, POU2F1, IL16		Regulation of transcription (TX)
GO:0042981	Regulation of apoptosis	**VEGFA, SPP1, BIRC5, CX3CR1, PLEKHF1, NLRP3, NR4A1,CDKN1A, BCL3, RIPK3, RAB27A**, ANGPTL4, IL2RB, ZBTB16, TCF7, SGMS1, JMY, TSC22D3, LTB		Apoptosis (AP)
GO:0006457	Protein folding	FKBP5, HSPH1, DNAJB13, HSP90AA1, DNAJA1, DNAJB1, FKBP4, AHSA1		Protein folding (PF)
GO:0033554	Cellular response to stress	**CHAF1A, CDK1, RAD51AP1, KIF22, CHAF1B, POLE, UHRF1, TIPIN, LIG1, CDKN1A, BCL3**, ANGPTL4, JMY, EPAS1		Stress response (SR)

Supplementary Table 3. Oligonucleotides used in real time PCR assays

Gene	Forward sequence	Reverse sequence
Vegfα	5'-AGCAGAAGTCCCATGAAGTGA-3'	5'-ATGTCCACCAGGGTCTCAAT-3'
Arg1	5'-GAATCTGCATGGGCAACCT-3'	5'-ACACGATGTCTTTGGCAGATAT-3'
Ym1	5′-GAAGGAGCCACTGAGGTCTG-3′	5′-GAGCCACTGAGCC TTCAAC-3′
Cd206	5'-TTCAGCTATTGGACGCGAGG-3'	5'-GAATCTGACACCCAGCGGAA-3'
Tgm2	5'-GGCCACTTCATCCTGCTCTA-3'	5'-TCCAAGGCACACTCTTGATG-3'
Il-10	5'-GGTTGCCAAGCCTTATCGGA-3'	5'-ACCTGCTCCACTGCCTTGCT-3
Angptl4	5'-ATGACTTCAGATGGAGGCTGG-3'	5'-AATTGGCTTCCTCGGTTCCC-3'
E2f1	5'-TTAGCCCTGGGAAGACCTCA-3'	5'-CCGTGGCAATACTGCTTCTTG-3'
CcnD1	5'-TCAAGTGTGACCCGGACTG-3'	5'-ATGTCCACATCTCGCACGTC-3'
CcnB2	5'-CCGACGGTGTCCAGTGATTT-3'	5'-CTGAGGTTTCTTCGCCACCT-3'
Cdk1	5'-ACACGAGGTAGTGACGCTGT-3'	5'-TCAATCTCTGAGTCGCCGTG-3'
Ube2c	5'-ATAGCCCTTTGAACACACACG-3'	5'-TGGAGACCTGCTTTGAATAGG-3'
Ki67	5'-AGAGCTAACTTGCGCTGACT-3'	5'-TCAATACTCCTTCCAAACAGGCA-3'
36B4	5′-GGCGACCTGGAAGTCCAACT-3	5′-CCATCAGCACCACGGCCTTC-3′

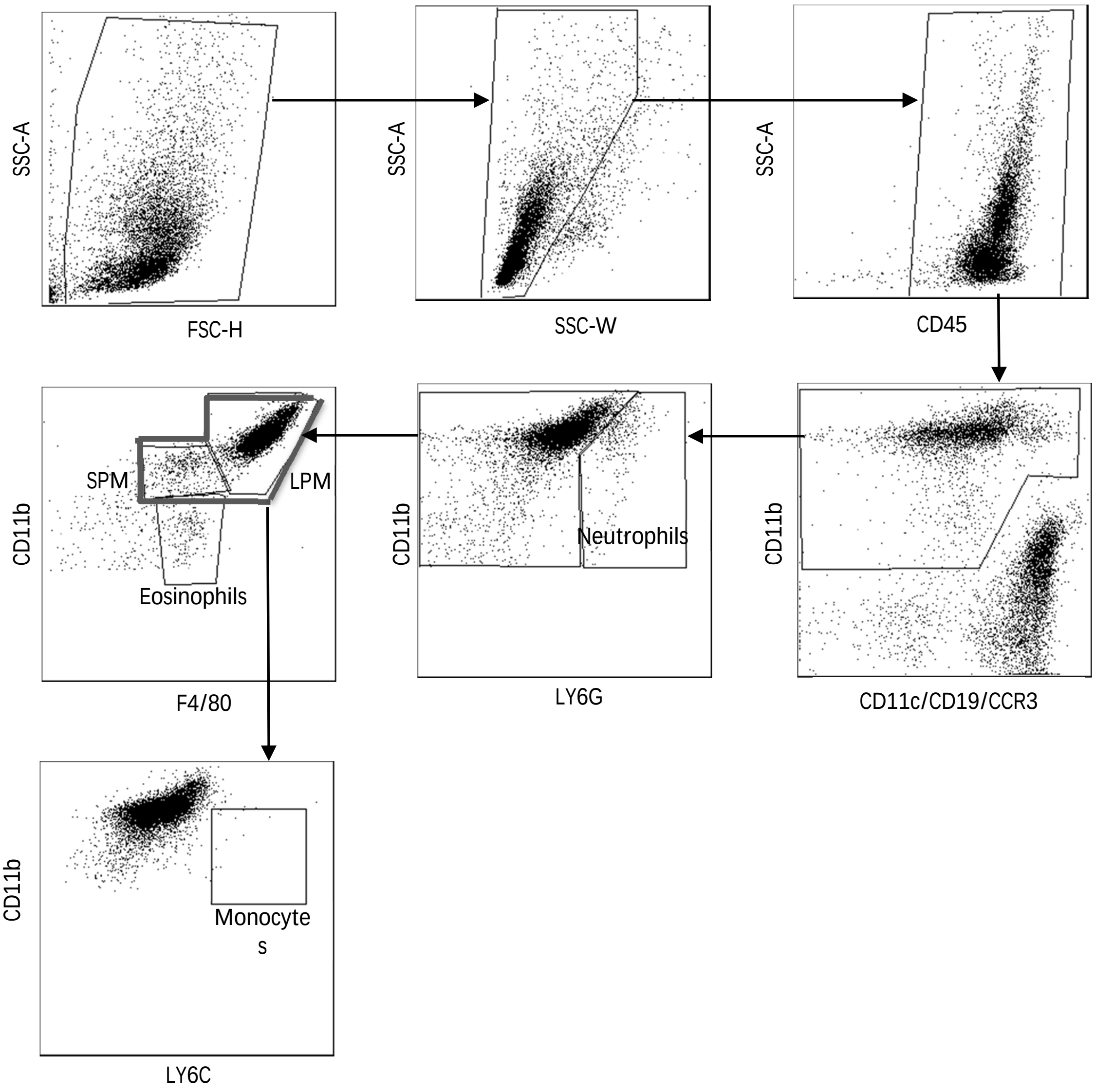

Supplementary Fig. 1

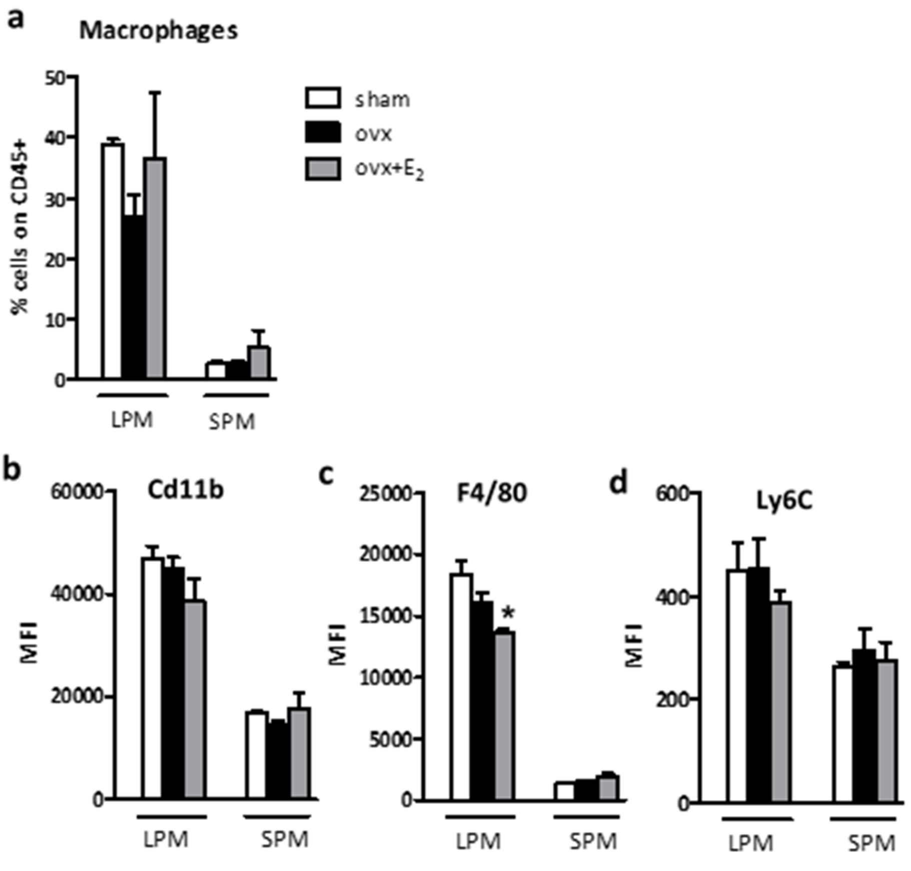

Supplementary Fig. 2

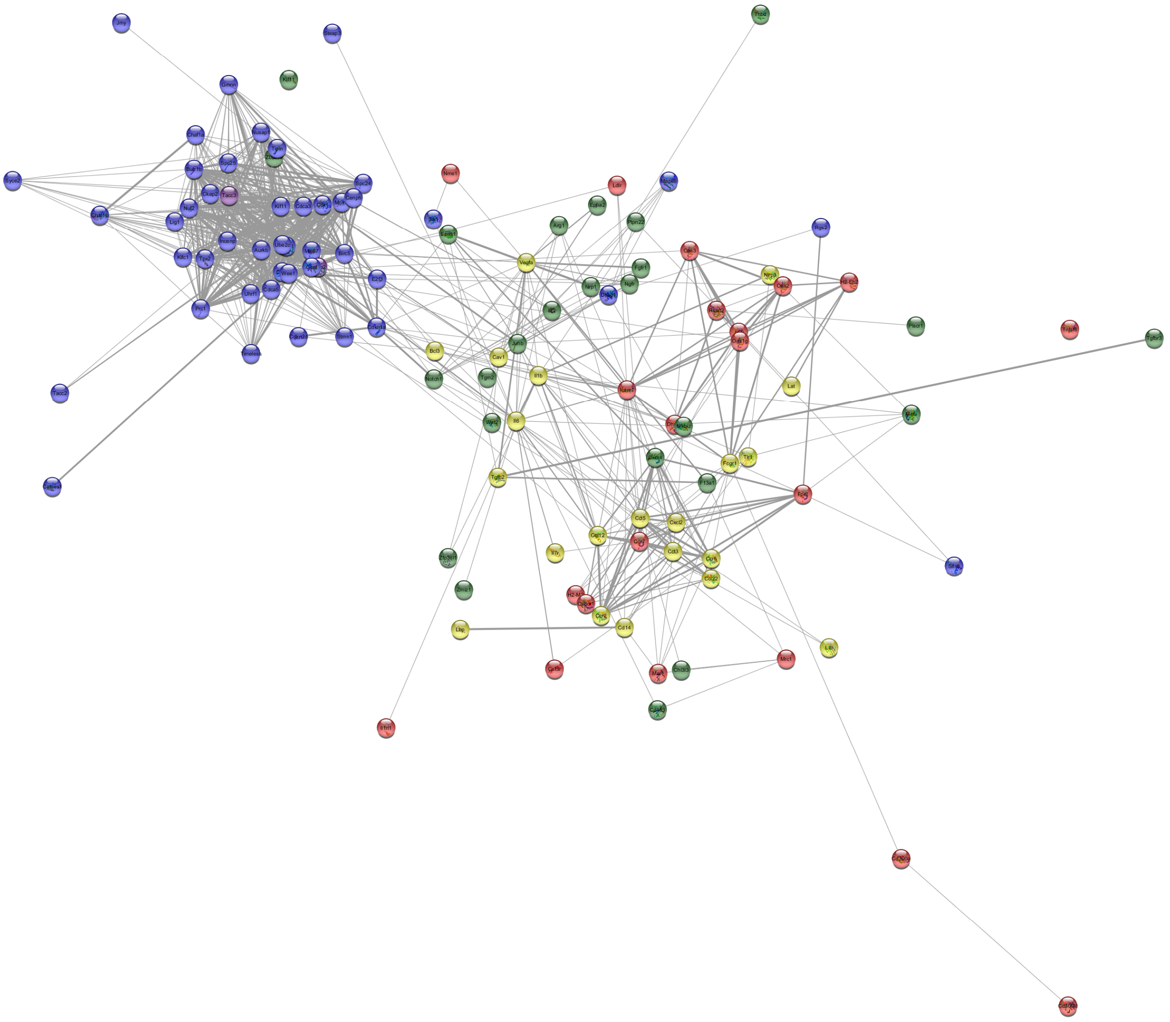

Supplementary Fig. 3

Supplementary Figure Legend

Supplementary Figure 1. Flow cytometry gating strategy of peritoneal macrophages.

Gating strategy for the identification by FACS analysis of neutrophils ($CD45^+$, $CD11c^-$,$CD19^-$,$CCR3^-$, $CD11b^+$, $LY6G^+$), eosinophils ($CD45^+$, $CD11c^-$,$CD19^-$,$CCR3^-$, $CD11b^{low}$, $LY6G^-$,$F4/80^{low}$), monocytes ($CD45^+$, $CD11c^-$,$CD19^-$,$CCR3^-$, $CD11b^+$, $LY6G^-$,$F4/80^+$, $LY6C^+$), small peritoneal macrophages (SPM; $CD45^+$, $CD11c^-$,$CD19^-$,$CCR3^-$, $CD11b^+$, $LY6G^-$,$F4/80^{low}$) and large peritoneal macrophages (LPM; $CD45^+$, $CD11c^-$, $CD19^-$, $CCR3^-$, $CD11b^+$, $LY6G^-$, $F4/80^{hi}$). One representative example for peritoneal cells from an unstimulated animal is shown.

Supplementary Figure 2. Flow cytometry analysis of peritoneal macrophages.

Large and small peritoneal macrophages (LPM and SPM, respectively) were identified by FACS analysis in the peritoneal lavage of sham (open bars), ovx (black bars) and ovx+E_2 (60 h treatment; grey bars) animals as described in Supplementary Figure 1. The percentage of LPM and SPM with respect to the total number of $CD45^+$ cells is reported in panel a, the expression level of CD11b, F4/80 and Ly6C are reported as Mean Fluorescence Intensity (MFI) in panels b to d, respectively. Results are expressed as the mean ± SEM (n=3). *$p < 0.05$ *versus* sham.

Supplementary Figure 3. String diagram of the interactions among estrogen regulated genes.

Estrogen regulated genes belonging to the Cell cycle (blue), Immune response (green) and Wound healing (red) pathways in peritoneal macrophages are evidenced, with commonly regulated genes resulting in purple (cell cycle+Immune response) and yellow (Immune response+Wound healing). This Figure is a higher resolution image of Figure 2b.
